# Evaluating the breast cancer predisposition role of rare variants in genes associated with low-penetrance breast cancer risk SNPs

**DOI:** 10.1186/s13058-017-0929-z

**Published:** 2018-01-09

**Authors:** Na Li, Simone M. Rowley, Ella R. Thompson, Simone McInerny, Lisa Devereux, Kaushalya C. Amarasinghe, Magnus Zethoven, Richard Lupat, David Goode, Jason Li, Alison H. Trainer, Kylie L. Gorringe, Paul A. James, Ian G. Campbell

**Affiliations:** 1 Cancer Genetics Laboratory, Research Division, Peter MacCallum Cancer Centre, 305 Grattan Street, Melbourne, VIC 3000 Australia; 2 Sir Peter MacCallum Department of Oncology, The University of Melbourne, Melbourne, VIC Australia; 3 Department of Pathology, Peter MacCallum Cancer Centre, Melbourne, VIC Australia; 4 Parkville Familial Cancer Centre, Peter MacCallum Cancer Centre and Royal Melbourne Hospital, Melbourne, VIC Australia; 5 LifePool, Peter MacCallum Cancer Centre, Melbourne, VIC Australia; 6 Bioinformatics & Cancer Genomics Laboratory, Peter MacCallum Cancer Centre, Melbourne, VIC Australia; 7 Bioinformatics Core Facility, Peter MacCallum Cancer Centre, Melbourne, VIC Australia; 8 Department of Pathology, University of Melbourne, Melbourne, VIC Australia; 9 Cancer Genomics Program, Peter MacCallum Cancer Centre, Melbourne, VIC Australia

**Keywords:** Familial breast cancer, Single-nucleotide polymorphism (SNP), Predisposition genes, Breast cancer susceptibility

## Abstract

**Background:**

Genome-wide association studies (GWASs) have identified numerous single-nucleotide polymorphisms (SNPs) associated with small increases in breast cancer risk. Studies to date suggest that some SNPs alter the expression of the associated genes, which potentially mediates risk modification. On this basis, we hypothesised that some of these genes may be enriched for rare coding variants associated with a higher breast cancer risk.

**Methods:**

The coding regions and exon-intron boundaries of 56 genes that have either been proposed by GWASs to be the regulatory targets of the SNPs and/or located < 500 kb from the risk SNPs were sequenced in index cases from 1043 familial breast cancer families that previously had negative test results for *BRCA1* and *BRCA2* mutations and 944 population-matched cancer-free control participants from an Australian population. Rare (minor allele frequency ≤ 0.001 in the Exome Aggregation Consortium and Exome Variant Server databases) loss-of-function (LoF) and missense variants were studied.

**Results:**

LoF variants were rare in both the cases and control participants across all the candidate genes, with only 38 different LoF variants observed in a total of 39 carriers. For the majority of genes (*n* = 36), no LoF variants were detected in either the case or control cohorts. No individual gene showed a significant excess of LoF or missense variants in the cases compared with control participants. Among all candidate genes as a group, the total number of carriers with LoF variants was higher in the cases than in the control participants (26 cases and 13 control participants), as was the total number of carriers with missense variants (406 versus 353), but neither reached statistical significance (*p* = 0.077 and *p* = 0.512, respectively). The genes contributing most of the excess of LoF variants in the cases included *TET2*, *NRIP1*, *RAD51B* and *SNX32* (12 cases versus 2 control participants), whereas *ZNF283* and *CASP8* contributed largely to the excess of missense variants (25 cases versus 8 control participants).

**Conclusions:**

Our data suggest that rare LoF and missense variants in genes associated with low-penetrance breast cancer risk SNPs may contribute some additional risk, but as a group these genes are unlikely to be major contributors to breast cancer heritability.

**Electronic supplementary material:**

The online version of this article (doi:10.1186/s13058-017-0929-z) contains supplementary material, which is available to authorized users.

## Background

Over the last decade, on the basis of genome-wide association studies (GWASs), > 100 common variants (single-nucleotide polymorphisms [SNPs]) have been reported to be associated with minor increases in breast cancer risk [1–3]. Researchers in fine-mapping studies have tried to identify the causal variants as a first step toward understanding how the elevated cancer risk is mediated. Nearly all of the SNPs are non-coding, and evidence to date suggests that some are in regulatory regions of neighbouring target genes and mediate subtle alterations in target gene expression, such as *CCND1* [4], or through changes in post-transcriptional regulation, such as altered splicing in *TERT* [5]. However, for most of the risk loci, the mechanism of risk modification has not been explained, although it is reasonable to expect that for many it will be through modifying expression or regulation of a target gene in the vicinity of the SNP. We hypothesised that if subtle expression changes confer a low susceptibility to breast cancer, coding variants in some of these genes might confer much higher levels of risk. This concept is supported by the finding of low-penetrance SNPs associated with known moderate- and high-penetrance genes such as *BRCA2*, *CHEK2* and potentially *RAD51B* (*RAD51L1*) [1–3], raising the possibility that other genes associated with low-penetrance SNPs might be enriched for coding high-penetrance predisposition alleles. To address this question, we sequenced all exons and exon-intron boundaries in 56 genes that are plausibly associated with breast cancer risk SNPs in index cases from 1043 familial breast cancer families who previously had negative test results for *BRCA1* or *BRCA2* pathogenic mutations and 944 population-matched cancer-free control participants from an Australian population.

## Methods

### Candidate genes

Because the target genes influenced by most reported breast cancer predisposition SNPs remain unknown, we used two strategies to identify genes of interest: (1) those reported as the plausible target gene in GWASs at the time of our gene panel design [2, 3, 6–13], and (2) where no gene had previously been proposed for a particular SNP, we screened any gene located ± 500 kb of the risk-associated SNP on the basis that most enhancers are < 500 kb away from the gene that they regulate and that most linkage disequilibrium (LD) blocks are < 500 kb in size [14]. In total, 56 genes associated with 56 SNPs were sequenced (Table 1, Additional file 1: Table S1), along with other candidates, as part of a custom sequencing panel [15–18].

**Table 1:** Candidate genes identified and corresponding breast cancer risk single-nucleotide polymorphisms

SNP	GWAS proposed candidates	Neighbouring genes ± 500 kb	SNP	GWAS proposed candidates	Neighbouring genes ± 500 kb
rs7726159	*TERT*	–	rs2016394	–	*DLX2*
rs10069690	*TERT*	–	rs1550623	*CDCA7*	–
rs2736108	*TERT*	–	rs6762644	–	*SETMAR*; *ITPR1*
rs2588809	*RAD51B*	–	rs12493607	*TGFBR2*	–
rs999737	*RAD51B*	–	rs9790517	*TET2*	–
rs10759243	–	*RAD23B*	rs6828523	*ADAM29*	–
rs2981579	*FGFR2*	–	rs1353747	*PDE4D*	–
rs11199914	–	*FGFR2*	rs1432679	*EBF1*	–
rs7072776	*DNAJC1*	–	rs204247	*RANBP9*	–
rs11814448	*DNAJC1*	–	rs720475	–	*TPK1*
rs13387042	–	*TNP1*	rs6472903	–	*HNF4G*
rs11552449	–	*DCLRE1B*	rs2943559	*HNF4G*	–
rs1045485	*CASP8*	–	rs7904519	*TCF7L2*	–
rs4973768	*SLC4A7*	–	rs3903072	–	*KAT5*; *SNX32*; *MUS81*
rs889312	*MAP3K1*	–	rs11820646	–	*NFRKB*
rs12662670	*ESR1*	–	rs2236007	*PAX9*	–
rs2046210	*ESR1*	–	rs941764	*CCDC88C*	–
rs1011970	*CDKN2A*; *CDKN2B*	–	rs17817449	*FTO*	–
rs704010	*ZMIZ1*	–	rs13329835	*CDYL2*	–
rs3817198	*LSP1*	–	rs527616	–	*CHST9*
rs10771399	*PTHLH*	–	rs1436904	*CHST9*	–
rs3803662	*TOX3*	–	rs4808801	*ELL*	–
rs6504950	*COX11*	–	rs3760982	–	*XRCC1*; *KCNN4*; *ZNF283*; *ZNF226*
rs8170	–	*USHBP1*; *BABAM1*; *UNC13A*	rs132390	–	*EMID1*; *NF2*
rs2363956	–	*USHBP1*; *BABAM1*; *UNC13A*	rs6001930	*MKL1*	–
rs2823093	*NRIP1*	–	rs4245739	*MDM4*	–
rs616488	*PEX14*	–	rs6678914	*LGR6*	–
rs4849887	–	*EPB41L5*	rs11075995	*FTO*	–

*GWAS* Genome-wide association study, *SNP* Single-nucleotide polymorphism

### Cohorts

A total of 1043 female breast cancer-affected index cases from high-risk breast cancer families were identified from the Variants in Practice Study and ascertained from familial cancer centres (FCCs) in Victoria and Tasmania, Australia, as described previously [17]. The personal and/or family history of all the cases were assessed by a specialist FCC and determined to be sufficiently strong to be eligible for clinical genetic testing for hereditary breast cancer predisposition genes by local criteria. All cases in this study had a negative test result for pathogenic mutations in *BRCA1* and *BRCA2*. The average age of cases in this study was 45 years (range, 22–81).

The control participants comprised 944 female subjects randomly selected from among the > 54,000 female participants of the Lifepool Study (http://www.lifepool.org/). The control participants had no self-reported or cancer registry-confirmed cancers diagnosed as of May 2016. Lifepool has recruited women > 40 years of age through the population-based mammographic screening program in Victoria, Australia (BreastScreen Victoria). The average age of Lifepool control DNA donors in this study was 59 years (range, 40–92).

### Targeted sequencing, variant calling and variant filtering

The coding regions and exon-intron boundaries (plus ≥ 10 bp of each intron) of 56 genes were enriched from germline DNA using a custom-designed HaloPlex Targeted Enrichment Assay panel (Agilent Technologies, Santa Clara, CA, USA). The libraries were sequenced on a HiSeq2500 Genome Analyzer (Illumina, San Diego, CA, USA) as described previously [17].

Sequencing data were processed and analysed using an in-house bioinformatics pipeline constructed using SEQLINER v0.1a (http://bioinformatics.petermac.org/seqliner). Raw reads (FASTQ files) were first quality-checked using FastQC (v0.11.2; http://www.bioinformatics.babraham.ac.uk/projects/fastqc/) and trimmed using cutadapt (1.7.1) [19] to ensure high read quality. Filtered reads were then aligned to the human reference genome (GRCh37/hg19) using the Burrows-Wheeler Aligner tool [20], with base quality score recalibration and indel realignment performed using the Genome Analysis Toolkit (GATK v3.2.2) [21]. GATK UnifiedGenotyper v2.4 (Broad Institute, Cambridge, MA, USA) [22], HaplotypeCaller [23] and PLATYPUS [24] were used for variant calling. Annotation of variants was performed using a local copy of the Ensembl [25] version R73 database and a customised version of Ensembl Variant Effect Predictor. Variants were determined by reference to the canonical transcripts. The Ensembl definition was as follows: (1) longest Consensus Coding Sequence Project translation with no stop codons; (2) if no (1), choose the longest Ensembl/Havana merged translation with no stop codons; (3) if no (2), choose the longest translation with no stop codons; (4) if no translation, choose the longest non-protein-coding transcript. Only variants that were identified by at least two variant callers with a total read depth of at least ten and an alternate allele read proportion ≥ 20% were included in the analysis. Loss-of-function (LoF) mutations were defined as stop-gained, frame shift or essential splice site mutations. The in silico assessment tools Condel [26], Polymorphism Phenotyping version 2 (PolyPhen-2) [27], SIFT [28], Combined Annotation Dependent Depletion (CADD) [29] and rare exome variant ensemble learner (REVEL) [30] were used to examine the likely pathogenicity of missense variants. Variant were defined as “likely deleterious” when predicted deleterious or damaging by Condel, PolyPhen-2 or SIFT, or when they had a CADD score ≥ 15 or a REVEL sore ≥ 0.5. The Exome Aggregation Consortium (ExAC) and Exome Variant Server (EVS) databases were used as additional references for the frequency of variants in the general population. Because this study was focused on the identification of moderate- to high-penetrance alleles, which will be rare [31, 32], only variants with a population allele frequency ≤ 0.001 (in both overall and European Caucasian populations) were assessed. Variants were visually inspected using Integrative Genomics Viewer [33, 34] to exclude artifacts.

### Statistical analysis

ORs and *p* values were calculated using a two-tailed Fisher’s exact test and the chi-square test in R version 3.3.2 [35].

## Results

All exons and exon-intron boundaries of 56 genes identified by either GWAS-proposed or location-based neighbouring criteria (Table 1; *see also* selection criteria described in the Methods section) were sequenced with consistent high coverage in cases and control participants (average sequencing depths of 170.4 and 175.6, respectively). Overall, 96.0% of the bases among the cases and 97.1% of the bases among the control participants were sequenced to a depth greater than tenfold (Additional file 1: Table S2). As previously described, principal component analysis using 7574 variants from all genes in the sequencing panel showed that ~ 98% of study subjects were of European Caucasian ancestry, and no bias was observed in the population distribution between the case and control cohorts [18].

### Loss-of-function variants

LoF variants (minor allele frequency [MAF] in ExAC and EVS, ≤ 0.001) were rare in both the cases and control participants across all the candidate genes, with only 38 unique variants observed in a total of 39 carriers (Table 2). For the majority of genes (36 of 56), no LoF variants were detected in either the case or control cohorts (Table 3).

**Table 2:** Loss-of-function variants detected in case and control cohorts

Symbol	CDS change^a^	Protein change	dbSNP identifier	Cases	Control participants	Consequence	EVS European MAF	ExAC non-Finnish European MAF
*ADAM29*	c.2020A > T	p.Lys674Ter	–	1	0	Stop-gained	0	0
*CASP8*	c.379C > T	p.Arg127Ter	–	1	0	Stop-gained	0	0
*CDKN2A*	c.225_243delCGCCACTCTCACCCGACCC	p.Ala76CysfsTer64	–	1	0	Frame shift	0	0
*CDKN2B*	c.149_150delCG	p.Ala50AspfsTer36	–	0	1	Frame shift	0	< 0.0001
*DCLRE1B*	c.189 + 1G > C		–	1	0	Splice donor	0	0
*DCLRE1B*	c.256G > T	p.Gly86Ter	–	0	1	Stop-gained	0	0
*FTO*	c.11delC	p.Pro5ArgfsTer13	–	1	0	Frame shift	0	0
*LGR6*	c.858-2A > C		–	1	0	Splice acceptor	< 0.0001	< 0.0001
*MUS81*	c.1314delC	p.Pro439LeufsTer6	–	1	0	Frame shift	0	0
*MUS81*	c.1062delC	p.Arg355GlyfsTer2	–	0	1	Frame shift	0	0
*NFRKB*	c.2149G > T	p.Glu717Ter	–	1	0	Stop-gained	0	0
*NFRKB*	c.794C > G	p.Ser265Ter	–	0	1	Stop-gained	0	< 0.0001
*NRIP1*	c.40_41insT	p.Asp14ValfsTer25	–	1	0	Frame shift	0	0
*NRIP1*	c.2750C > G	p.Ser917Ter	–	1	0	Stop-gained	0	0
*NRIP1*	c.1968dupT	p.Gly657TrpfsTer5	–	1	0	Frame shift	0	0
*PDE4D*	c.2400_2410dupTGTCATAGATG	p.Asp804ValfsTer3	–	1	0	Frame shift	0	0
*RAD51B*	c.139C > T	p.Arg47Ter	rs200355697	2	0	Stop-gained	0	0.0001
*SETMAR*	c.823_826delAAAG	p.Glu276GlyfsTer2	–	1	0	Frame shift	0	< 0.0001
*SETMAR*	c.706C > T	p.Arg236Ter	–	0	1	Stop-gained	0	0.0001
*SETMAR*	c.1635C > G	p.Tyr545Ter	–	0	1	Stop-gained	0	0
*SLC4A7*	c.1663G > T	p.Gly555Ter	–	1	0	Stop-gained	0	0
*SNX32*	c.1111C > T	p.Arg371Ter	–	1	0	Stop-gained	0	< 0.0001
*SNX32*	c.825 + 2 T > G		–	1	0	Splice donor	0	0
*TCF7L2*	c.1804_1805insAAT	p.Glu602_Glu603insTer	–	0	1	Stop-gained	0	0
*TET2*	c.1085_1086insT	p.Pro363SerfsTer6	–	1	0	Frame shift	0	0
*TET2*	c.2072delC	p.Thr691MetfsTer9	–	1	0	Frame shift	0	0
*TET2*	c.3646C > T	p.Arg1216Ter	–	1	0	Stop-gained	0	0
*TET2*	c.4361_4362insG	p.Arg1455GlnfsTer23	–	1	0	Frame shift	0	0
*TET2*	c.3812_3820delGCGCCTGTC	p.Cys1271_Gln1274delinsTer	–	1	0	Stop-gained	0	0
*TET2*	c.832C > T	p.Gln278Ter	–	0	1	Stop-gained	0	0
*TET2*	c.1458delC	p.Asn486LysfsTer11	–	0	1	Frame shift	0	0
*TPK1*	c.185 + 1G > A		–	0	1	Splice donor	0	0
*USHBP1*	c.1220 + 1G > T		rs144791770	1	0	Splice donor	0.0002	0.0001
*USHBP1*	c.258dupA	p.Val87SerfsTer103	–	0	1	Frame shift	0.0001	< 0.0001
*ZNF226*	c.1229_1230delAA	p.Arg411SerfsTer11	–	1	0	Frame shift	0	0.0001
*ZNF226*	c.2239C > T	p.Arg747Ter	–	1	0	Stop-gained	0	0
*ZNF226*	c.2380G > T	p.Glu794Ter	rs201830106	0	1	Stop-gained	0.0007	0.0003
*ZNF226*	c.582delT	p.Asn194LysfsTer41	–	0	1	Frame shift	0	0

*Abbreviations*: *CDS* Coding DNA sequence, *EVS* Exome Variant Server, *ExAC* Exome Aggregation Consortium, *MAF* Minor allele frequency, *dbSNP* Single-nucleotide polymorphism database

^a^Canonical transcript for each gene according to Ensembl definition

**Table 3:** Number of carriers with loss-of-function and missense variants detected in case and control cohorts

Gene	Selection criteria	Number of carriers with loss-of-function variants					Number of carriers with missense variants				
		Case	Control	*p* Value^a^	OR	95% CI	Case	Control	*p* Value^a^	OR	95% CI
*TET2*	GWAS proposed	5	2	0.456	2.27	0.37–23.87	20	18	1	1.01	0.50–2.03
*NRIP1*	GWAS proposed	3	0	0.251	Und	0.37–∞	21	17	0.632	1.12	0.56–2.28
*RAD51B*	GWAS proposed	2	0	0.501	Und	0.17–∞	6	4	0.756	1.36	0.32–6.57
*SNX32*	Neighbouring genes	2	0	0.501	Und	0.17–∞	3	6	0.323	0.45	0.07–2.12
*ZNF226*	Neighbouring genes	2	2	1	0.91	0.07–12.5	24	18	0.640	1.21	0.63–2.39
*ADAM29*	GWAS proposed	1	0	1	Und	0.02–∞	13	11	1	1.07	0.44–2.65
*CASP8*	GWAS proposed	1	0	1	Und	0.02–∞	8	2	0.113	3.64	0.72–35.26
*CDKN2A*	GWAS proposed	1	0	1	Und	0.02–∞	3	3	1	0.91	0.12–6.77
*DCLRE1B*	Neighbouring genes	1	1	1	0.91	0.01–71.08	7	6	1	1.06	0.30–3.82
*FTO*	GWAS proposed	1	0	1	Und	0.02–∞	10	11	0.668	0.82	0.31–2.14
*LGR6*	GWAS proposed	1	0	1	Und	0.02–∞	16	8	0.217	1.82	0.73–4.94
*MUS81*	Neighbouring genes	1	1	1	0.91	0.01–71.08	8	9	0.808	0.80	0.27–2.36
*NFRKB*	Neighbouring genes	1	1	1	0.91	0.01–71.08	17	12	0.577	1.29	0.58–2.97
*PDE4D*	GWAS proposed	1	0	1	Und	0.02–∞	6	3	0.512	1.81	0.39–11.24
*SETMAR*	Neighbouring genes	1	2	0.607	0.45	0.01–8.70	7	3	0.349	2.12	0.48–12.73
*SLC4A7*	GWAS proposed	1	0	1	Und	0.02–∞	14	10	0.682	1.27	0.52–3.21
*USHBP1*	Neighbouring genes	1	1	1	0.91	0.01–71.08	14	11	0.841	1.15	0.48–2.82
*CDKN2B*	GWAS proposed	0	1	0.475	0	0–35.30	1	1	1	0.91	0.01–71.08
*TCF7L2*	GWAS proposed	0	1	0.475	0	0–35.30	5	8	0.406	0.56	0.14–1.96
*TPK1*	Neighbouring genes	0	1	0.475	0	0–35.30	2	2	1	0.91	0.07–12.50
*ZNF283*	Neighbouring genes	–	–	–	–	–	17	6	0.057	2.59	0.97–8.06
*HNF4G*	GWAS proposed	–	–	–	–	–	4	1	0.377	3.63	0.36–178.82
*TERT*	GWAS proposed	–	–	–	–	–	5	6	0.765	0.75	0.18–2.97
*UNC13A*	Neighbouring genes	–	–	–	–	–	17	8	0.158	1.94	0.79–5.21
*LSP1*	GWAS proposed	–	–	–	–	–	11	15	0.327	0.66	0.27–1.55
*XRCC1*	Neighbouring genes	–	–	–	–	–	6	12	0.153	0.45	0.14–1.30
*ZMIZ1*	GWAS proposed	–	–	–	–	–	15	11	0.694	1.24	0.53–3.00
*EMID1*	Neighbouring genes	–	–	–	–	–	11	8	0.654	1.25	0.46–3.59
*FGFR2*	GWAS proposed	–	–	–	–	–	4	4	1	0.91	0.17–4.87
*CCDC88C*	GWAS proposed	–	–	–	–	–	38	45	0.219	0.76	0.47–1.20
*ITPR1*	Neighbouring genes	–	–	–	–	–	17	20	0.507	0.77	0.37–1.55
*MKL1*	GWAS proposed	–	–	–	–	–	26	19	0.547	1.24	0.66–2.40
*CHST9*	GWAS proposed	–	–	–	–	–	7	9	0.617	0.70	0.22–2.13
*PEX14*	GWAS proposed	–	–	–	–	–	9	6	0.613	1.36	0.43–4.66
*PAX9*	GWAS proposed	–	–	–	–	–	3	7	0.207	0.39	0.06–1.70
*PTHLH*	GWAS proposed	–	–	–	–	–	3	1	0.626	2.72	0.22–142.85
*CDCA7*	GWAS proposed	–	–	–	–	–	5	3	0.729	1.51	0.29–9.76
*MAP3K1*	GWAS proposed	–	–	–	–	–	20	11	0.206	1.66	0.75–3.85
*RANBP9*	GWAS proposed	–	–	–	–	–	10	5	0.309	1.82	0.56–6.80
*DNAJC1*	GWAS proposed	–	–	–	–	–	8	9	0.808	0.80	0.27–2.36
*TOX3*	GWAS proposed	–	–	–	–	–	7	7	1	0.90	0.27–3.03
*EPB41L5*	Neighbouring genes	–	–	–	–	–	8	8	1	0.90	0.29–2.78
*ESR1*	GWAS proposed	–	–	–	–	–	3	6	0.323	0.45	0.07–2.12
*MDM4*	GWAS proposed	–	–	–	–	–	7	3	0.349	2.12	0.48–12.73
*CDYL2*	GWAS proposed	–	–	–	–	–	11	5	0.217	2	0.64–7.37
*TNP1*	Neighbouring genes	–	–	–	–	–	0	2	0.226	0	0–4.82
*BABAM1*	Neighbouring genes	–	–	–	–	–	4	3	1	1.21	0.20–8.27
*TGFBR2*	GWAS proposed	–	–	–	–	–	4	3	1	1.21	0.20–8.27
*ELL*	GWAS proposed	–	–	–	–	–	9	5	0.430	1.63	0.49–6.23
*NF2*	Neighbouring genes	–	–	–	–	–	12	5	0.150	2.19	0.71–7.95
*KCNN4*	Neighbouring genes	–	–	–	–	–	8	4	0.393	1.82	0.49–8.27
*DLX2*	Neighbouring genes	–	–	–	–	–	7	4	0.553	1.59	0.40–7.42
*KAT5*	Neighbouring genes	–	–	–	–	–	3	2	1	1.36	0.16–16.29
*COX11*	GWAS proposed	–	–	–	–	–	2	1	1	1.81	0.09–106.93
*EBF1*	GWAS proposed	–	–	–	–	–	2	3	0.673	0.60	0.05–5.27
*RAD23B*	Neighbouring genes	–	–	–	–	–	0	1	0.475	0	0–35.30
GWAS proposed genes	–	17	4	0.008	3.89	1.26–15.95	287	251	0.679^b^	1.05	0.86–1.28
Neighbouring genes	–	9	9	1	0.90	0.32–2.58	168	138	0.392^b^	1.12	0.87–1.44
Total	–	26	13	0.077	1.83	0.90–3.90	406	353	0.512^b^	1.07	0.89–1.28

Abbreviations: GWAS Genome-wide association study, *Und* undefined

^a^Fisher’s exact test, two-sided

^b^Pearson’s chi-square test with the Yates correction

No gene had a significant excess of LoF mutations in the cases versus the control participants. *TET2* had the largest number of LoF variants, with five in the cases and two in the control participants, whereas three LoF mutations were detected in *NRIP1* but none in the control participants. No more than two mutation carriers were identified in each cohort for the remaining 18 genes harbouring LoF variants. Across all 56 genes, there was a total 26 LoF mutations in the cases compared with 13 among the control participants (OR, 1.83; *p* = 0.077; 95% CI, 0.9–3.9). Notably, there were ten genes with LoF variants detected only in the cases, compared with only three genes with LoF variants detected only in the control participants. Restricting this analysis to only the 35 genes directly proposed by GWASs with a potentially higher likelihood of being the target gene (as opposed to being based solely on their location ± 500 kb from the SNP), we observed a significant excess of LoF mutations in the cases (17 versus 4; OR, 3.89; 95% CI, 1.26–15.95; *p* = 0.008). In contrast, no difference was observed for the 21 location-only-based candidate genes (9 versus 9).

### Missense variants

Similar to the LoF variants, the total number of carriers with rare missense variants (MAF ≤ 0.001 in ExAC and EVS) (Table 3, Additional file 1: Table S3) across all 56 genes was greater in the cases than in the control participants (406 versus 353; OR, 1.07), but this finding was not statistically significant (*p* = 0.512). In addition, 34 genes had a higher frequency of missense variants in the cases compared with only 16 genes with a higher frequency in the control participants. *ZNF283* showed the strongest enrichment for missense variants in the cases (17 versus 6); however, this difference was not statistically significant. There was no obvious difference in the rare missense variant frequency based on whether they were GWAS-proposed genes or location-only-based genes.

The missense variants were further stratified according to a series of in silico prediction tools (Condel, PolyPhen-2, SIFT, CADD and REVEL) as a means of enriching for variants with a higher likelihood of pathogenicity (Table 4). There was a trend towards a slightly higher frequency of predicted pathogenic missense variants observed in the cases than in the control participants using any single prediction tool (ORs ranging from 1.11 to 1.37), but none of the comparisons reached statistical significance. Further restricting the analysis to only those variants predicted to be pathogenic by all five in silico tools, we detected no significant difference between the cases and the control participants (58 versus 39; *p* = 0.170).

**Table 4:** Number of carriers with likely deleterious missense variants predicted by in silico tools

Rare missense variants (MAF ≤ 0.001)	Number of carriers		Number of total subjects		*p* Value^a^	OR	95% CI
	Cases	Control participants	Cases	Control participants			
All	406	353	1043	944	0.512	1.07	0.89–1.28
Condel deleterious	174	136	1043	944	0.182	1.19	0.93–1.53
PolyPhen-2 Probably/possibly deleterious	198	164	1043	944	0.384	1.11	0.88–1.41
CADD score ≥ 15	225	173	1043	944	0.08	1.23	0.98–1.54
SIFT deleterious	171	131	1043	944	0.134	1.22	0.94–1.57
REVEL score ≥ 0.5	88	63	1043	944	0.163	1.29	0.91–1.83
Predicted deleterious by all	58	39	1043	944	0.170	1.37	0.89–2.13

*Abbreviations*: *CADD* Combined Annotation Dependent Depletion, *MAF* Minor allele frequency, *PolyPhen-2* Polymorphism Phenotyping version 2, *REVEL* Rare exome variant ensemble learner

^a^Pearson’s chi-square test with the Yates correction

## Discussion

The majority of common, low-penetrance breast cancer SNPs are located in non-coding genomic regions, and although different hypotheses have been proposed, the biological mechanisms underlying these risk associations remain inconclusive. Studies to date have demonstrated mechanisms at least for some risk SNPs involving altered expression of the target gene as a result of disruption to enhancer or promoter regions or by affecting RNA splicing [4, 5]. On this basis, we hypothesised that if subtle alterations to gene expression result in small increases in breast cancer risk, then coding variants with more profound effects on gene function might convey much higher levels of risk. *BRCA1* and *BRCA2* are the prime examples of such a scenario where both highly penetrant coding mutations and low-penetrance non-coding SNPs exist. GWASs are not designed to identify such variants, owing to their rarity in the population.

Among the 56 candidate genes sequenced, LoF variants were rare, with over half of genes having no LoF variants in either the cases or control participants. However, there was a small excess of both the total number of LoF and missense variants in the cases compared with the control participants (LoF OR, 1.83; missense OR, 1.07), but because the mutation frequency for each individual gene was very low, it is unclear if this result reflects a higher penetrance effect of a small number of genes or if many of the variants contributed to a small excess in breast cancer risk. The genes with the greatest contribution to the excess of LoF variants in the cases included *TET2*, *NRIP1*, *RAD51B* and *SNX32* (12 cases versus 2 control participants), whereas *ZNF283* and *CASP8* contributed largely to the excess of missense variants (25 cases versus 8 control participants). However, on an individual gene level, none showed a significant difference in the cases compared with the control participants. A larger cohort size is needed to confirm this trend and identify the contribution of any single gene. Of note, there were no LoF variants detected and no excess of missense variants (four in cases versus four in control participants) in *FGFR2*, the “top hit” in many independent breast cancer GWASs.

The strongest excess of LoF variants in this study was *TET2* (five cases versus two control participants). This gene was reported to have a genome-wide influence on gene expression by altering DNA methylation whereby its dysregulation was associated with aberrant DNA methylation and involved in the development of acute myeloid leukaemia [36, 37]. Guo et al. showed that the association with cancer appeared to be with functional SNPs that lie in the promoter or enhancer that consequently affects *TET2* expression [38]. Such evidence suggested that it is plausible that rare coding variants in *TET2* could lead to compromised TET2 function and involvement in breast cancer susceptibility. However, the data for *TET2* need to be interpreted cautiously because it is a gene known to cumulate age-related somatic mutations in blood [39]. It is possible that some of the variants we identified are somatic mutations rather than germline variants, particularly in light of the fact that the alternate allele read proportions of LoF variants were generally in the low range (≤ 35%).

Researchers have proposed that LoF variants in *RAD51B* (*RAD51L1*) confer a high risk of breast cancer [40], but it remains inconclusive owing to the extreme rarity of the LoF mutations (only 48 carriers in 60,706 participants in ExAC; carrier frequency, 0.08%). Few germline LoF mutations have been reported: one splicing variant in a breast and ovarian cancer family [41], one splicing and one nonsense variant in two patients with ovarian cancer [42], and one nonsense variant in a melanoma family (p.Arg47Ter) [43]. We observed two carriers of the same nonsense mutation, p.Arg47Ter, which is the most common LoF variant seen in ExAC database (21 carriers in total, including 14 South Asian and 5 non-Finnish European carriers). In addition to breast cancer family history, each carrier had a relative with ovarian cancer (mother, grandmother), and one had both parents diagnosed with melanoma. Together with the previously cited reports, our data support *RAD51B* as a plausible candidate gene in breast cancer families, especially breast and ovarian cancer families, and it may also play a role in melanoma predisposition.

With respect to missense variants, *CASP8* showed a strong signal towards an excess of rare variants (eight cases versus two control participants). Notably, the corresponding low-penetrance GWAS SNP rs1045485 (p.Asp344His; MAF_ExAC_, 0.12) is a missense variant in *CASP8*; however, it is not included in the missense variants in this study, because we focused only on the rare variants (MAF, ≤ 0.001). In a meta-analysis of one promoter polymorphism that decreased *CASP8* expression, Cai et al. concluded that it was associated with a reduced risk of a broad range of cancers, including breast cancer [44]. This evidence and our data would be consistent with a model whereby a subtle reduction in CASP8 function leads to reduction in cancer risk, whereas missense mutations conferring an enhanced or altered function increase cancer risk. Regardless of the status of these leading candidate genes, our data clearly show that low-penetrance SNP-associated genes are not conspicuously enriched for high-penetrance breast cancer predisposition alleles and at best could explain only a small proportion of hereditary breast cancer families with no known pathogenic variants.

It has been suggested that one possible mechanism contributing to the minor risks detected in GWASs for common variants that lie close to the coding sequence of a gene could be an uneven distribution of much rarer, high-risk coding variants between the different SNP alleles. For many SNPs this explanation appears unlikely on the basis of underlying LD structure and the distance between the tagging SNP and the nearest gene, and for a smaller number this has been excluded by fine-mapping and functional studies that have directly demonstrated the effect of the causative variant. However, our data provide an opportunity to examine this potential mechanism systematically for all of the genes sequenced. We compared the frequency with which LoF and rare missense variants in the 56 genes were observed in association with either the corresponding risk SNP or the alternate allele, both in the case group and in the control group (Additional file 1: Table S4), and we found no convincing evidence of an interaction between the common and rare variants. For a few genes, including *PDE4D* and *TERT*, there was a notable trend towards an excess of rare variants in association with the risk form of the SNP, but this was not statistically significant when adjusted for the effect of multiple testing. Similar trends were observed for some genes, including *UNC13A* and *DNAJC1*, in the opposite direction, indicating that the trends on each side of the association were very likely due to random chance. Of note, the greatest excess of rare variants in carriers of the risk allele was found for the *PDE4D* gene, where pathogenic missense variants have previously been associated with an unrelated rare high-penetrance dominant disorder, acrodysostosis type 2 [45].

This study has several main limitations. Firstly, as a consequence of the rarity with which LoF variants were observed in these candidate genes, our cohort size could not provide sufficient power to determine the cancer predisposition role for any individual gene. Secondly, further breast cancer predisposition SNPs continue to be identified, and we have not analysed genes that are located near more recently identified SNPs, although there is no reason to believe that the genes we studied are not representative of SNP-related genes in general. Thirdly, the cases and control participants in this analysis are well matched for ethnicity and represent a very similar population in which the predisposition SNPs were originally identified. However, we are unable to evaluate if moderate- to higher-penetrance predisposing variants do exist in other ethnic groups. In addition, in this study, we were not able to examine whether some candidate genes were significant in specific molecular subtypes of breast cancer.

## Conclusions

In summary, our study describes, for the first time to our knowledge, an assessment of the contribution of rare coding variants in SNP-associated genes to familial breast cancer risk. Although confirmatory studies are required, our data suggest that rare LoF and missense variants in genes associated with low-penetrance SNPs may contribute some additional risk but that they are unlikely to be major contributors to breast cancer heritability.

## Additional file

Additional file 1: Table S1. Genome coordinates and reported ORs for the breast cancer risk SNPs used in this study. **Table S2.** Sequencing coverage of 56 candidate genes in case and control cohorts. **Table S3.** Rare (MAF, < 0.001) missense variants detected in case and control cohorts. **Table S4.** SNP and rare variant association analysis. (XLSX 111 kb)

## Abbreviations

CADD: Combined Annotation Dependent Depletion
CDS: Coding DNA sequence
EVS: Exome Variant Server
ExAC: Exome Aggregation Consortium
FCC: Familial cancer centre
GWAS: Genome-wide association study
LD: Linkage disequilibrium
LoF variant: Loss-of-function variant
MAF: Minor allele frequency
PolyPhen-2: Polymorphism Phenotyping version 2
REVEL: Rare exome variant ensemble learner
SNP: Single-nucleotide polymorphism
